# Contextual drivers on local sport promotion policy: a Dutch case study on the relevance of the COVID-19 pandemic

**DOI:** 10.1080/19406940.2024.2378143

**Published:** 2024-07-16

**Authors:** Linda Ooms, Remco Hoekman

**Affiliations:** a Department of Sociology, Mulier Institute, Utrecht, The Netherlands; b Department of Sociology, Radboud University, Nijmegen, The Netherlands

**Keywords:** COVID-19, local sport promotion policy, local sport manager, neighbourhood sport coach, multiple streams framework, socioecological model

## Abstract

The aim of this study was to explore why and how the COVID-19 pandemic influenced local sport promotion policy in the Netherlands. We used a case study approach examining the Dutch context. We studied the perspectives of Local Sport Managers (LSMs) and (coordinators of) neighbourhood sport coaches (NSCs). We applied the principles of the Multiple Streams Framework (MSF) within a socioecological model of local sport promotion policy to examine whether the COVID-19 pandemic created a ‘policy window’ for policy change and if so why. Online questionnaires were sent to LSMs (*n* = 352, response 26%) and online group interviews (*n* = 11) were conducted with LSMs and (coordinators of) NSCs. Our study revealed the COVID-19 pandemic indeed opened a ‘policy window’ and resulted in local policy changes related to the facilities and public space (hardware), organisational actors (orgware) and organised activities (software). In most cases, this did not lead to totally new policy, but more a shift in focus. National politics as a response on the COVID-19 pandemic increased the generic awareness of the importance of sport for health. This helped in legitimising the existence of sport promotion policy for a broader purpose. It is important for LSMs and NSCs to take advantage of this ‘policy window’ and use it to create advocacy and support for sport promotion policy over a longer period. As policy change and implementation normally take a period of multiple years, the current Dutch policy framework provides (financial) opportunities for LSMs and NSCs to follow this through.

## Introduction

It is well known that regular participation in sport and physical activity is important for the health of people of all ages. It decreases the risk for chronic diseases and mortality and enhances mental health (Physical Activity Guidelines Advisory Committee 2018, World Health Organisation (WHO) 2020, Eather *et al*. 2023). Moreover, participation in sport and physical activity with others is associated with improved social functioning and social well-being (Andersen *et al*. 2018, Eather *et al*. 2023). Especially, in the COVID-19 pandemic these advantages were highlighted and brought to the front. Being sufficiently physically active during the pandemic was, for example, associated with less depressive symptoms, stress, loneliness and insomnia (Meyer *et al*. 2020, Lewis *et al*. 2021, Reyes-Molina *et al*. 2022). Furthermore, people who were regularly active had a lower risk of COVID-19 infection and a lower likelihood of adverse COVID-19 outcomes (Sallis *et al*. 2021, Ezzatvar *et al*. 2022).

Although governments were aware of these benefits and emphasised the importance of staying physically active during the COVID-19 pandemic, the COVID-19 restrictions (e.g. closure of sport facilities, social distancing, cancellation of competitions) have impacted greatly on the sport sector and the possibilities for people to be physically active. Sport organisations faced new challenges in engaging and maintaining sport participants, coaches, and volunteers, managing finances, and creating meaningful and socially interactive sport activities (Grix *et al*. 2020, Elliot *et al*. 2021, Staley *et al*. 2021). Furthermore, studies in the Netherlands and other countries showed a general decrease in sport and physical activity behaviour among inhabitants during the COVID-19 pandemic (De Boer *et al*. 2021, Grubben *et al*. 2022, Widyastari *et al*. 2022, Wilke *et al*. 2022). Also, there are signals that the COVID-19 pandemic magnified existing inequalities in sport and physical activity participation, particularly, for people with low incomes, financial problems, low education and the unemployed (De Boer *et al*. 2021, Grubben *et al*. 2022, Kyan and Takakura 2022, Widyastari *et al*. 2022). It is suggested that these subgroups have in general fewer resources (e.g. social contacts, knowledge, money) to participate in sport and physical activity and, accordingly, to adapt to the COVID-19 restrictions (Grubben *et al*. 2022). Moreover, it is not self-evident that people who decreased or even stopped their sport and physical activity behaviour, will automatically return to their old habits on their own (Elliot *et al*. 2021). Acknowledging the layered perspective of local sport promotion policy, being situated in and impacted by a broader context (Fahlén and Stenling 2016, Vos *et al*. 2016, Hoekman and Scheerder 2021, Hoekman *et al*. 2022), this raises the question whether these developments due to the COVID-19 pandemic have influenced local policy makers’ decisions regarding their sport promotion policies.

This question is theoretically linked to the Multiple Streams Framework (MSF) in which a crisis can be seen as a trigger or ‘policy window’ in which policy entrepreneurs must be ready to promote their problems and solutions. Policy formation can be seen as the result of the interplay of three sets of processes or streams (problems, policies and politics) that are coupled or connected by policy entrepreneurs (Kingdon 1995).

The MSF has been widely applied by sport policy researchers for the analyses of policy stability and change (e.g. Houlihan and Green 2006, Sotiriadou and Brouwers 2012, Camargo *et al*. 2020, Jayawardhana and Piggin 2021, Jedlicka *et al*. 2022). These studies, however, mostly focus on the national policy landscape. With regard to the local sport policy landscape research is scarce. In general, local sport promotion policy is characterised by a limited critical reflexivity (Mansfield 2016, Hoekman *et al*. 2022). As such, there is limited information on how local sport promotion policy adapts to changing environments, such as the natural experiment of the COVID-19 pandemic. All in all, little research has focused on contextual drivers of local sport promotion policy and, consequently, a generic theoretical framework for local sport policy analysis is lacking (Hoekman and Scheerder 2021, Hoekman *et al*. 2022). Furthermore, the MSF is criticised for paying limited attention to contextual drivers (Jayawardhana and Piggin 2021, Jedlicka *et al*. 2022). Therefore, we combine a more contextual socio-ecological approach (Hoekman and Scheerder 2021, Hoekman *et al*. 2022) with the principles of the MSF to develop a more holistic approach of local sport promotion policy analysis.

The central question of our study is to examine whether the COVID-19 pandemic influenced local sport promotion policy and, in particular, the attention for sport promotion of specific population subgroups. We will use a case study approach examining the Dutch context. Hereby, we will focus on the experiences of Local Sport Managers (LSMs) and Neighbourhood Sport Coaches (NSCs), who can be seen as ‘policy entrepreneurs’ of local sport promotion policy development in the Netherlands. Our study addresses two research questions: 1) Which developments in the surrounding conditions due to the COVID-19 pandemic, as perceived by the LSMs and NSCs, led to changes in local sport promotion policy (*why* question)? 2) How did local sport promotion policy change due to these developments in the surrounding conditions (*how* question)?

To answer these research questions, we use a holistic approach, by integrating the MSF principles within a socioecological model of local sport promotion policy development. Furthermore, we use a nationally grounded model to describe the different elements of local sport promotion policy (i.e. hardware, orgware, software; Association of Sport and Municipalities 2018). With this, we aim to provide more insight into how local sport promotion policy is developed and adapts to changing circumstances. We pay attention to the accompanying policy processes and how local sport promotion policy gets meaning and is legitimatised as an area of local public policy, in the context of the COVID-19 pandemic and growing social inequalities. So, with this research we add to the existing body of knowledge about local sport promotion policy development in a broader (changing) context and to the theoretical advancement on this subject.

## Dutch sport promotion policy context and the COVID-19 pandemic

In the Netherlands, as in many European countries, responsibility for sport promotion policy lies primarily at the local level. However, unlike other European countries, local sport promotion policy is not mandated by any law (Hoekman and Breedveld 2013). Sport promotion services are mainly delivered by municipalities, at the municipalities’ discretion and paid for from municipal budgets. LSMs are the heads of municipal sport policy departments and are responsible for sport promotion policy development within a municipality (Hoekman *et al*. 2022). The NSCs are, as front-line professionals, the most important implementers of local sport promotion policy activities, with tasks including facilitating collaboration between the sport sector and other sectors (e.g. education, welfare), referring inhabitants to appropriate sport and physical activities and organising sport and physical activities. Moreover, they often provide important input to the LMSs with regard to their local sport promotion policies. In 328 of 344 Dutch municipalities NSCs are employed (Heijnen *et al*. 2022). So both the LSMs and the NSCs can be seen as important ‘policy entrepreneurs’. They are involved in both sport policy agenda setting and in finding and creating appropriate solutions to important issues or problems related to sport.

The COVID-19 pandemic caused an obvious policy window and provided opportunities for policy changes. In most Dutch municipalities, NSCs kept, in the first year after the introduction of the COVID-19 pandemic, focussing on stimulating sport and physical activity among their target groups and, due to the closure of sports clubs and indoor sport facilities, there was an increased focus on enhancing sport and physical activity in public space. However, because of the COVID-19 measures, more than a quarter of the NSCs were less active in organising activities (Ooms 2021, Ooms and Van Stam 2021).

Nonetheless, a majority of LSMs reported that the COVID-19 pandemic did not lead to more attention on addressing social inequalities in sport and physical activity participation and additional or changed sport promotion policy measures in the first year of the pandemic (Ooms 2021). This is to an extent understandable as the first year is characterised by uncertainty about the COVID-19 pandemic and the need to constantly adapt to new or changing COVID-19 measures. However, one would anticipate that two years after the start of COVID-19 measures more structural policy changes become visible and it is this period that constitutes the subject of this study.

## Theoretical framework

In our case, the COVID-19 pandemic and related COVID-19 measures provided a context in which local sport promotion policy was situated. To understand how local sport promotion policy changed in this policy window of COVID-19, we used a case study design focusing on the Dutch context. A case study approach is an appropriate methodological design to capture the complexity of the local sport promotion policy process and its broader context (Fahrner and Klenk 2018). In addition, to unravel this complexity, empirical mixed-methods studies are required (Evans 2020), which are scarce in sport policy research (Grix *et al*. 2018). Therefore, we used multiple methods which enables complementary analysis to address various aspects in the policy process (Cairney 2012). In line with Pielke and Harris (2021), we consider methods and theories as intellectual tools and frameworks that allow us to construct a conceptual map of a particular policy context and gain insight in how changes have come about regarding local sport promotion policy.

Our study addresses the level of local authorities as being the most important and most tangible governmental sport structure to promote sport participation close to the target groups (Vos *et al*. 2016, Hoekman *et al*. 2022). We used the MSF as our guiding theoretical framework (Kingdon 1995, Jayawardhana and Piggin 2021, Jedlicka *et al*. 2022). The MSF distinguishes three streams: problems, policies and politics. Problems can be identified as issues that need to be addressed, such as the decreased sport and physical activity levels due to the COVID-19 pandemic. Policies are the solutions, such as the stimulation of sport and physical activity in public space during the COVID-19 pandemic. Politics are the political reasons why certain issues and solutions may be selected, such as available financial resources, dominant policy paradigms (e.g. sport for all vs. the instrumental values of sport) and existing laws and policy measures (e.g. the national implemented COVID-19 measures). The MSF assumes that policy changes occur when these three streams converge and create a ‘policy window’. This means there is a defined problem, an acceptable solution and the political will to do something. A policy window could be triggered by an external focusing event, such as the COVID-19 crisis. Furthermore, ‘policy entrepreneurs’ play an important role in coupling of a certain problem with a solution and convincing policy makers to support this problem/policy solution. If this succeeds, policy change occurs.

We integrated the principles of the MSF in a socioecological model to conceptualise the different environmental systems that influence local sport promotion policy (Bronfenbrenner 1979, Hoekman and Scheerder 2021). With this, we address a general critique on the MSF that the attention on the context is rather limited (Jayawardhana and Piggin 2021, Jedlicka *et al*. 2022). Furthermore, we used a nationally grounded model to describe the different elements of sport promotion policy, i.e. hardware, orgware and software (Association of Sport and Municipalities 2018). Although the latter model is based on the Dutch context, the terms hardware, orgware and software are also internationally accepted (e.g. Hoekman 2023). In general, it is anticipated that for an optimal sport promotion policy and social value it is vital that the hardware (the sport facilities and spaces), orgware (the organisational actors and human capital) and software (organised sport activities and interventions) are attuned to the population and the policy goals. Furthermore, we enriched our model with the perspectives of other studies focussing on aspects of local sport promotion policy in the Netherlands (Hoogendam *et al*. 2021, Hoekman *et al*. 2022) and on (local) sport promotion policy (analysis) in other countries (Houlihan 2005, Fahlén and Stenling 2016, Vos *et al*. 2016, Stenling and Sam 2017).

**Figure 1.**
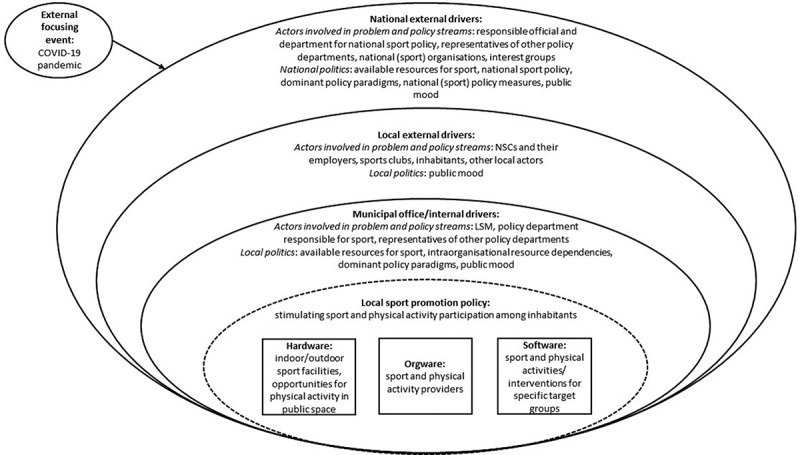
Theoretical framework of local sport promotion policy in the Netherlands.

Our theoretical framework is presented in Figure 1. Different parts of the framework will be conceptualised below starting with the sport promotion policy which is central in this model. Next, we describe the different environmental systems and factors within that system that influence sport promotion policy (i.e. contextual drivers) and how they are related to the MSF principles. We start with the closest environment being within the municipal offices (internal drivers) and end with national level drivers.

### Local sport promotion policy

The main aim of local sport promotion policy is to stimulate sport participation (both organised and non-organised) and physical activity (e.g. at home, in public space) among inhabitants. ‘Sport for all’ (see also Council of Europe 2021) is a central theme of local sport promotion policy in the Netherlands, with a specific focus to include groups that lag behind in sport and physical activity participation. However, there is also an apparent shift noticeable from access to sport as a social right, in line with the ‘Sport for all’ philosophy, to ‘sport as a means’, that is, sport as an instrument for addressing a wider range of (policy) issues, such as improving health, social participation and integration (Hoogendam *et al*. 2021, Hoekman *et al*. 2022). This shift is largely influenced by the ‘healthification’ of Dutch national sport policy (Stuij and Stokvis 2015; see also: *national external drivers*).

Local sport promotion policy can influence sport and physical activity participation through three elements or pillars: 1) improving the physical infrastructure for sport and physical activity by providing indoor and outdoor sport facilities and opportunities to be physically active in public space (*hardware*); 2) facilitating and supporting sport and physical activity providers, such as sports clubs and schools (*orgware*) and 3) facilitating or implementing sport and physical activities and interventions for specific target groups (*software).*

### Municipal office/internal drivers

The municipal office is an organisational entity consisting of different policy departments. Different actors may be involved in the problem and policy streams at this level: a LSM is responsible for sport promotion policy development and can be seen as the policy entrepreneur in this regard. However, the policy development is also dependent on the department configuration within which sport promotion policy is situated (e.g. within sport, public health, welfare), and the collaboration the LSM has with other policy departments (e.g. public health, education) within the municipality (Houlihan 2005, Hoekman *et al*. 2022). In the past years, collaboration between LSMs and other policy departments increased, especially to promote the instrumental values of sport (Hoekman *et al*. 2022). Local politics may influence the attention for and content of sport promotion policy as well, such as the available resources for sport, intraorganisational resource dependencies (e.g. access to financial resources, expertise), dominant policy paradigms and the public mood (e.g. sport is good for health) (Houlihan 2005, Fahlén and Stenling 2016, Hoekman *et al*. 2022).

### Local external drivers

Local sport promotion policy development is the result of the efforts of actors within and outside municipal offices. Local actors outside the municipal offices are increasingly involved in the problem and policy streams of local sport promotion policy. This holds true for both demand groups and providers of sport and physical activities. Local actors may contribute with their opinions and solutions to the public mood, i.e. the local politics.

The way local actors are involved differs between municipalities and has changed over the years (Vos *et al*. 2016, Hoogendam *et al*. 2021). In the Netherlands, the municipality has mostly the role of co-producer. This means local sport promotion policy is developed and implemented by a LSM in collaboration with NSCs and their employers (often local sport services, but also schools, sports clubs, welfare organisations), and to a lesser extent, voluntary sports clubs, inhabitants, and other local organisations. Another vital role of the municipality is that of coordinator. In this case, the LSM formulates the aims and preconditions for sport promotion policy and NSCs and other local actors are responsible for realising these (Hoogendam *et al*. 2021).

### National external drivers

Although local sport promotion policy is developed and implemented at the local level, it can be influenced by national external drivers, such as national politics. National politics consists of, for example, the influence of national (sport) organisations and interest groups (e.g. Association of Sport and Municipalities) on the public mood, the vision and ideas of the responsible official and department for national sport policy development and the national sport policy itself (Houlihan 2005, Fahlén and Stenling 2016, Stenling and Sam 2017, Hoekman *et al*. 2022). National sport policy is the responsibility of the minister of Health, Welfare and Sport. Regarding national sport policy programmes, the Broad Regulation for Combination functions (BRC) and the National Sports Agreement are important. Within the BRC, the Dutch government will provide 73.3 million euros per year to employ NSCs within municipalities (i.e. 40% of the employment is funded by the state and 60% by the municipality or other local organisations) over the period of 2019–2022 (Heijnen *et al*. 2022). In addition, the National Sports Agreement holds importance for the local level as it urges the development of local sports agreements in which the municipality, local sport and physical activity providers and other local organisations, collaborate to make plans to stimulate sport and physical activity participation in the municipality. An important theme is ‘inclusive sport and physical activity’ which is aimed to take away barriers for sport and physical activity participation for less active and vulnerable population groups. The Dutch government financially supports the local sports agreements (Dutch Ministry of Health, Welfare and Sport 2018b). Both national programmes reflect the ‘Sport for all’ philosophy as well as the instrumental values of sport. Over the past decades, health-related aims gained dominance in Dutch national sport policy, the so-called ‘healthification’ of sport (Stuij and Stokvis 2015). Consequently, sport got a more prominent role in other policy domains, such as health and physical environment (Dutch Ministry of Health, Welfare and Sport 2018a, Ruijgt *et al*. 2018). Dominant core policy paradigms and discourses at the national level visibly influence sport promotion policy topics at the local level (Houlihan 2005; Fahlén and Stenling 2016, Hoekman and Scheerder 2021, Hoekman *et al*. 2022; see also: *local sport promotion policy*). So might the COVID-19 policy measures implemented by the national government.

### External focusing events

Different external focusing events, such as an economic recession, natural disasters, climate change and the COVID-19 pandemic, might influence local sport promotion policy (in)directly through the different drivers (internal and external) mentioned before (Houlihan 2005, Parnell *et al*. 2018, Hoekman *et al*. 2022). In this article, our focus is on the COVID-19 pandemic.

So, in sum, coupling our theoretical framework with our research questions, the first research question aims to reveal developments in the surrounding conditions due to the COVID-19 pandemic as presented by the external and internal drivers in our theoretical framework (*why* question). It is anticipated that the external focusing event of the COVID-19 pandemic will influence the surrounding contextual drivers of sport promotion policy with an emphasis on the instrumental value of sport. Considering the decreased physical activity levels in society of specific population groups and the already induced shift to a focus on the instrumental values of sport, we expect that the COVID-19 pandemic will further enhance this shift and create a favourable (policy) environment for sport promotion in general and sport promotion policy in particular.

The second research question aims to capture the changes in the local sport promotion policy itself (*how* question) instigated by the external and internal drivers and represented by changes in the hardware, orgware and software. If indeed, as we anticipate, the focus on the instrumental values of sport will be enhanced, we expect changes in the hardware, orgware and software side of the sport promotion policy. With regards to the hardware, we anticipate a stronger focus on physical activity friendly environments and opportunities for sport and physical activity in public space. Considering the orgware, the support for local voluntary sports clubs is worth mentioning as the instrumental value of sport is partly linked to sports club participation. With respect to the software of local sport promotion policy, more (focus on) sport and physical activities and interventions for specific target groups is anticipated.

## Methods

This study used a mixed-methods design, combining both quantitative and qualitative research methods. The quantitative part particularly focused on the ‘how’ question and consisted of an online questionnaire among LSMs in the Netherlands. It was considered an explorative study which informed the topics of the qualitative part. The qualitative part focused both on the ‘why’ and ‘how’ questions and consisted of in-depth online group interviews with LSMs and (coordinators of the) NSCs.

### Quantitative explorative study

An online questionnaire was open from 1 December 2021 until 26 January 2022 and send to LSMs in the panel of LSMs of the Association of Sport and Municipalities^1^ which represents all municipalities in the Netherlands (*n* = 352). In December 2021, there was a general lockdown in the Netherlands, with most public facilities, including sports clubs, being closed. In January 2022, the lockdown was cancelled. Outdoor sport participation was possible without any restrictions. For indoor sport facilities, a COVID-19 digital passport was mandated.

The questionnaire consisted of closed and open questions. First, some background questions were asked, i.e. questions about the existence of a local sports agreement; the importance of sport promotion as a policy theme; and sport promotion policy’s target groups. The final two questions mainly focused on the ‘how’ question of this study, namely the extent to which the COVID-19 pandemic influenced sport promotion for target groups; and whether the COVID-19 pandemic led to structural changes in sport promotion policies. In addition, the LSM was asked whether he or she was willing to participate in an online group interview, also including other important developers of the local sport promotion policy (when relevant) and the implementers of the local sport promotion policy within their municipality. After a reminder, a total of 91 LSMs filled in the questionnaire (response 26%). The data were analysed using SPSS software. The descriptive results are presented, whereby the data have been weighted by urbanity level of the municipality. With this, the data are representative of all municipalities in the Netherlands.

### Online group interviews

To assess LSMs and NSCs subjective views and experiences on the consequences of the COVID-19 pandemic on local sport promotion policy in more detail, online in-depth semi-structured group interviews were performed. The group interviews were performed for each municipality separately and in a sequential way. In-depth interviewing is appropriate to address why and how questions and to understand the interviewees’ perceptions of processes, decision-making, motivations, and expectations regarding the topic (Weiss 1994, Guest *et al*. 2013).

Of the questioned LSMs in the quantitative study, twenty-one (23%) were willing to participate in an online group interview. For this study, 11 municipalities were selected. This means there were 11 group interviews in total. Sampling was purposeful in the sense that a diversity of municipalities was captured regarding population size, geographical location, and the answers the LSMs provided in the online questionnaire (e.g. with regard to the influence of the COVID-19 pandemic on local sport promotion policy). For more information on the characteristics of the municipalities, see Table 1. Although the sample is relatively small, additional information was reduced in progressing towards the eleventh interview. This led us to conclude that data saturation had been reached.

**Table 1.** Characteristics of the municipalities participating in the online group interviews.

Municipality number (changes in local sport promotion policy due to COVID-19*)	Region in the Netherlands (north, middle, south)	Population size	Sport policy document (published)	Local sports agreement (published)	Number of interviewees (including LSM)
1 (yes)	South	<25.000	No	Yes (May 2020), aims are leading for sport promotion policy	1
2 (yes)	North	≥75.000	No, but they are now developing a combined sport and health policy	Yes (June 2020)	3
3 (no)	North	25.000–75.000	No, but there are concrete assignments for the NSCs	Yes (2020)	2
4 (yes)	Middle	≥75.000	Yes (2017)	Yes (February 2020)	3
5 (yes)	Middle	≥75.000	Yes (March 2021)	No, aims of local sports agreement are integrated in general sport promotion policy	1
6 (yes)	North	25.000–75.000	Yes (November 2019)	Yes (July 2020)	2
7 (yes)	North	25.000–75.000	Yes (March 2021)	Yes (Spring 2020)	2
8 (don’t know)	Middle	25.000–75.000	Yes (January 2022)	Yes (April 2020)	2
9 (don’t know)	Middle	25.000–75.000	Yes (Winter 2021)	Yes (2020)	2
10 (don’t know)	North	25.000–75.000	Yes (March 2022)	Yes (January 2020)	2
11 (don’t know)	North	25.000–75.000	Yes (March 2021)	Yes (June 2020)	2

*Based on questionnaire data.

The LSMs were invited by email to participate in an online group interview. In addition, the LSM was asked to invite other persons that were also responsible for the local sport promotion policy development (when relevant) and the most important implementers of local sport promotion policy activities. For almost all municipalities, a NSCs and/or a coordinator of the NSCs (i.e. a person who leads a group of NSCs) joined the interview and for two municipalities also a colleague policy maker working within the same policy department was present. All interviewees signed a digital informed consent form before participation. Participants were not exposed to procedures, nor were they obligated to follow certain behavioural rules. Therefore, in accordance with the Dutch Medical Research Involving Human Subjects Act (WMO), medical ethics committee’s approval was not necessary for conducting this study (Dutch Government 2023).

The group interviews consisted of 1–3 persons (see also Table 1). Of the interviewees, 36% were female. They had an average age of 43 years (range 29–63 years) and were working on average 6 years (range 8 months-20 years) in their function as a policy maker or policy implementer (not in Table).

The group interviews were conducted by the first author between April 2022 and June 2022 using the online platform Teams. At that moment, there were no COVID-19 restrictions in the Netherlands. Before performing the interview, the researcher requested the sport promotion policy document when available. This document was read to get familiar with the local sport promotion policy, its aims and target groups, and possible influences of the COVID-19 pandemic (when mentioned) on the policy. This helped in conducting the interview and ensured all aspects of the policy were discussed. Interview questions were built around how sport promotion policy is developed and who engages in development and implementation; the content of the sport promotion policy; whether the COVID-19 pandemic and measures influenced sport promotion policy and if so why and how. Hereby, also the results of the online questionnaire were used as input. The open-ended questions facilitated informal conversations with the interviewees about their knowledge and experiences about the topics questioned. In addition, it provided options for the researcher to follow leads and uncover prominent issues as they arose.

The group interviews lasted on average 1,5 hours and were digitally recorded. The interviews were transcribed verbatim. MAXQDA version 2022 was used for data analysis. Data analysis was performed by the first author. Thematic analysis (Nowell *et al*. 2017) was performed to examine the perspectives of the interviewees concerning the influence of the COVID-19 pandemic and measures on local sport promotion policy. The analysis was done for the ‘why’ and ‘how’ questions separately. Hereby, the following steps were performed: first, data were analysed deductively based on the research questions and theoretical framework. When new themes emerged, new codes were inductively added. To enhance trustworthiness of the data analyses, the steps for thematic analyses as recommended by Nowell *et al*. (2017) were followed. This involved repeated reading of the transcripts with the framework and research questions in mind to identify patterns, similarities, and differences between municipalities. To enhance credibility and trustworthiness, the analysis was continuously discussed with the second author.

## Results

This section describes the results of the online questionnaire and group interviews. The findings are arranged as follows. First, we will present general results about local sport promotion policies and influences of the COVID-19 pandemic on these policies (particularly the ‘how’ question). Second, based on the group interviews, we will present more in-depth information on the influence of the COVID-19 pandemic on external and internal drivers and related changes in local sport promotion policy (the ‘why’ and ‘how’ question). This is presented separately for hardware, orgware and software. One of the main findings was that very little variation was found between the municipalities that participated in the group interviews.

### General results: local sport promotion policy

The results of the online questionnaire showed that 94% of Dutch municipalities consider sport promotion as an important or very important policy theme. In addition, 95% of Dutch municipalities have a local sports agreement including the theme ‘inclusive sport and physical activity’. This means there is specific attention on sport promotion of population groups that lag behind in sport and physical activity participation (see Table 2). The same holds true for the municipalities that participated in the online group interviews (see Table 1).

**Table 2.** Results closed questions online questionnaire among LSMs.

Question	Percentage
How important is sport promotion as a policy theme within your municipality? (*n* = 91)	
Very important	43
Important	51
Neutral	4
Unimportant	0
Very unimportant	0
Don’t know	1
Not applicable, sport promotion is no policy theme	2
Does your municipality have a local sports agreement? (*n* = 91)	
Yes, including theme ‘inclusive sport and physical activity’	95
Yes, but without theme ‘inclusive sport and physical activity’	4
No	0
Don’t know	1
Do you think the COVID-19 pandemic has structural consequences for yourmunicipality’s sport promotion policy? (*n* = 89)	
Yes	55
No	25
Don’t know	19

Based on the group interviews, it is shown that the LSM developed the sport promotion policy in collaboration with other policy domains (e.g. health, welfare, education). In eight of the 11 participating municipalities, the department of sport was situated in a broader policy domain (e.g. social domain, sport and education), which enhanced intersectoral collaboration. As a result, sport promotion policy aims are often part of a broader vision, which is also visible in the names of the sport promotion policy documents, such as ‘*a healthy, happy and vital [name municipality]*’ and ‘*a vision on living together 2020–2024*’. Also, other stakeholders provided input for the sport promotion policy, most often (coordinators of) NSCs, and, to a lesser extent, sports clubs, other local organisations and inhabitants. Next to NSCs as most important implementers of sport promotion policy activities, sports clubs were also often mentioned as important implementers.

### General results: influence of COVID-19 pandemic and measures

**Figure 2.**
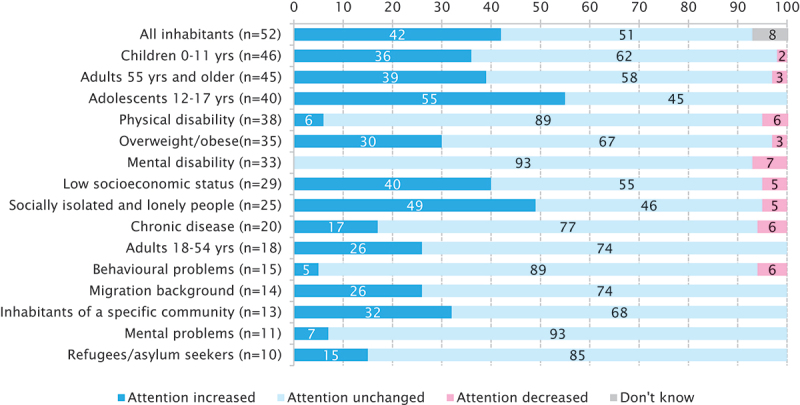
Target groups* of local sport promotion policy and changes in attention for these target groups due to the COVID-19 pandemic.

The results of the online questionnaire showed that the COVID-19 pandemic has led to increased attention for sport promotion of different target groups, in particular adolescents (55% of LSMs who aim at this target group say the attention for this group has increased), socially isolated and lonely people (49%) and people with a low socioeconomic status (40%). However, a large part of LSMs report the attention for their target group(s) did not change (see Figure 2).

* The most frequently mentioned target groups are placed in the upper part of the figure.

In addition, the results of the online questionnaire indicated that 55% of the LSMs think the COVID-19 pandemic will lead to structural changes in their local sport promotion policy (see Table 2). Based on the open answers, four important topics emerged: ‘using sport for prevention of diseases/to promote health’, ‘an increased focus on less active target groups’, ‘increased support of sports clubs’ and ‘more promotion of sport in public space’ (not in Table). However, 25% think the COVID-19 pandemic will not lead to structural changes in their local sport promotion policy because attention is already paid to inactive target groups and/or the belief that after the pandemic ends everything will get back to normal again (see also Table 2).

In the group interviews, which were held after COVID-19 measures were cancelled, these results were further explored. The results of the thematic analyses show that the COVID-19 pandemic influenced external and internal drivers which impacted the local sport promotion policy (see Table 3). In all participating municipalities, the COVID-19 pandemic led to changes in local sport promotion policy (although respondents at first indicated in the questionnaire that this was not the case). The *why* and *how* will be described in more detail below. This will be done separately for hardware, orgware and software.

**Table 3.** Results thematic analyses online group interviews: why and how local sport promotion policy changed due to the COVID-19 pandemic.*

	Reasons for changes in local sport promotion policy due to developments in the surrounding conditions (why)		
Changes in local sport promotion policy (how)	Municipal office/internal drivers	Local external drivers	National external drivers
**Hardware**			
More (attention for) sport facilities in public space	Increased experience of the importance of sport in public space by LSMs and other policy domains (local politics) More collaboration between LSM and other policy domains (e.g. urban planning) (solution)	Limited sport possibilities (problem) Finding alternative sport possibilities in public space by local sport providers, NSCs and inhabitants (solution) Increased sport participation in public space (solution) Increased experience of the importance of sport in public space by local sport providers, NSCs and inhabitants (local politics)	*Implementation of COVID-19 measures by the national government, specifically the forced closure of indoor and outdoor sport facilities* (national politics) Increased experience of the importance and stimulation of sport participation for promoting health/prevention of disease (e.g. prevention of COVID-19) by the national government (national politics) Increased experience of the importance and stimulation of sport participation in public space by the national government (national politics)
**Orgware**			
Enhanced contacts and collaboration with sports clubs and other local sport providers	Temporary different role of LSM: clarifying measures and supporting sport providers with applying for financial compensation arrangements (solution)	Limited sport possibilities (problem) Finding alternative sport possibilities by local sport providers (solution) Uncertainties about what is allowed and what is not considering sport offer (problem) Confusion about temporary financial compensation arrangements by sport providers (problem) Temporary different role of NSC: clarifying measures and supporting sport providers with applying for financial compensation arrangements (solution)	*Implementation of different and quickly changing COVID-19 measures for the sport sector (e.g. closure of sport facilities, social distancing rules) by the national government* (national politics) Provision of temporary financial compensation arrangements for sports providers by the national government (national politics)
Increased support for sports clubs	Awareness of acceleration of problems facing sports clubs due to the COVID-19 measures by LSMs (problem) Increased experience of the importance of role sports clubs in promoting health (physical, mental, and social health) by LSMs (local politics)	Limited sport possibilities (problem) Finding alternative sport possibilities by inhabitants (solution) Decreases in members of sports clubs, particularly team and indoor sports (problem) Decreases in volunteers at sports clubs (problem) Increased experience of the importance of role sports clubs in promoting health (physical, mental, and social health) by local actors (including the NSCs and inhabitants) (local politics)	*Implementation of COVID-19 measures by the national government, specifically the forced closure of sports clubs, social distancing rules* (national politics) Increased experience of the importance and stimulation of sport participation for promoting health/prevention of disease (e.g. prevention of COVID-19) at home and in public space by the national government (national politics)
Enhanced collaboration with non-sport organisations (e.g. health and youth welfare organisations, schools)	Increased experience of the importance of sport participation for promoting health (physical, mental, and social health) by LSMs and other policy domains (local politics) More connection between LSM and other policy domains (e.g. health, welfare, youth) (solution)	Increased experience of the importance of sport participation for promoting health (physical, mental, and social health) by local actors (local politics) Enhanced (re)connection between sport and non-sport organisations (solution) Enhanced (re)connection between NSCs and non-sport organisations (solution)	Increased experience of the importance and stimulation of sport participation for promoting health/prevention of disease (e.g. prevention of COVID-19) by the national government (national politics)
**Software**			
More sport activities (using sport as a means) for specific target groups, mainly children, adolescents, and older adults	Increased experience of the importance of sport participation for promoting health (physical, mental, and social health) by LSMs and other policy domains (local politics) Increased experience of the physical, mental, and social health problems with specific population subgroups (problem) More collaborative sport initiatives (i.e. sport as a means) between LSM and other policy domains (e.g. health, welfare, youth) (solution) Reflecting on target groups for sport promotion policy (solution)	Increased experience of the importance and stimulation of sport participation for promoting health (physical, mental, and social health) (local politics) Increased experience of the physical, mental, and social health problems with specific population subgroups by local actors (problem) New collaborative sport initiatives by NSCs, sport and non-sport organisations (solution)	*Implementation of different COVID-19 measures by the national government, specifically the lock downs, social distancing, and home quarantining rules* (national politics) Increased experience of the importance and stimulation of sport participation for promoting health (physical, mental, and social health)/prevention of disease (e.g. prevention of COVID-19) by the national government (national politics) Increased experience of the physical, mental, and social health problems with specific population subgroups by the national government (problem) Implementation of temporary funding arrangements (COVID-19 recovery funds) by the national government to stimulate social and mental wellbeing and a healthy lifestyle among youth, vulnerable groups, and lonely older adults (national politics)
More (support of) sport activities in public space, particularly for youth	Increased experience of the importance of sport participation for promoting health (physical, mental, and social health) by LSMs and other policy domains (local politics) Increased experience of the importance of sport in public space by LSMs and other policy domains (local politics) Increased experience of the physical, mental, and social health problems with youth (problem) More collaborative outdoor sport initiatives (i.e. sport as a means) between LSM and other policy domains (e.g. health, welfare, youth), particularly for youth (solution)	Limited sport possibilities (problem) Finding alternative sport possibilities in public space by local sport providers, NSCs and inhabitants (solution) Increased sport participation in public space (solution) Increased experience of the importance of sport in public space by local sport providers, NSCs and inhabitants (local politics) Increased experience of the physical, mental, and social health problems with youth by local actors (problem) New collaborative outdoor sport initiatives by NSCs, sport and non-sport organisations, particularly for youth (solution)	*Implementation of COVID-19 measures by the national government, specifically the forced closure of indoor and outdoor sport facilities* (national politics) Increased experience of the importance and stimulation of sport participation for promoting health/prevention of disease (e.g. prevention of COVID-19) by the national government (national politics) Increased experience of the importance and stimulation of sport participation in public space by the national government (national politics) Increased experience of the physical, mental, and social health problems with youth by the national government (problem) Implementation of temporary funding arrangements (COVID-19 recovery funds) by the national government to stimulate social and mental wellbeing and a healthy lifestyle among youth, vulnerable groups, and lonely older adults (national politics)

* COVID-19 measures are in *italic*. De different streams (problem, policy/solution, politics) from the MSF are placed between (…).

### Changes in sport facilities and public space (hardware)

One common topic emerged from the group interview data regarding changes in hardware: 1) More (attention for) sport facilities in public space.

#### More (attention for) sport facilities in public space

The COVID-19 pandemic resulted in different policy actions and vision on how to combat the pandemic. At the national level, the Dutch government implemented different measures to reduce the spread of the virus. These also included the closure of indoor and outdoor sport facilities, including sports clubs and gyms, for a longer period. Concurrently, the national government emphasised the importance of staying physically active to remain healthy, because there were indications that a healthy and active lifestyle could prevent serious disease from COVID-19 infection. However, due to the installed measures, people were advised to do sport at home or in public space.

These national politics influenced local level actors. At the local level, sport providers, NSCs and inhabitants were forced to find alternative sport possibilities and, consequently, there was an increase in use of public space for sport by inhabitants (solutions). This made the importance of public space for sport more visible at all levels (national and local politics):

Well, I think the awareness of the importance of public space for sport has increased overall, from the national level to the local level, but also within the municipal office itself: among the council, the mayor and aldermen support for this [theme] increased. – LSM average size municipality

However, this does not mean that sport in public space was not a policy theme before the COVID-19 pandemic. The pandemic made it more urgent, and this urgency was also felt by other policy domains within the municipal office:

I think that public space and being physically active close to home, running in a green environment, the importance of that function of the environment increased. And it also helps us with our colleagues of urban planning, who are more responsible for that [policy] part. We have now much more contact with them [than before the pandemic] and they are thinking with us about how a green city can contribute to health. – LSM large size municipality

Considering changes in hardware, the experienced urgency resulted in concrete policy aims related to better and more sport facilities in public space, sometimes in collaboration with the urban planning policy domain. In addition, it accelerated existing plans, such as the building of a physical activity route with ‘playground equipment’ for older adults around a health care centre. In this way, people could exercise outdoors by themselves during the pandemic.

### Changes in organisational actors (orgware)

From the interview data, three common topics emerged regarding changes in orgware: 1) Enhanced contacts and collaboration with sports clubs and other local sport providers; 2) Increased support for sports clubs; and 3) Enhanced collaboration with non-sport organisations.

#### Enhanced contacts and collaboration with sports clubs and other local sport providers

At the national level, the Dutch government implemented different but also quickly changing COVID-19 measures for the sport sector (national politics). At the local level, these caused confusion and uncertainties among sport providers about what was allowed and what was not (problem). Also, the compensation funds for sport providers (national politics: national compensation funds for lost income or rental costs) installed by the national government led to many questions by local sport providers (problem). Consequently, NSCs (at the local level) and LSMs (within the municipal office) got a temporary different role translating the COVID-19 restrictions to the local context and helping sports providers with applying for funding (temporary solution):

During the COVID-19 period, I was contact person of the municipality for all sports clubs. So, I was very busy with handling this task. Sometimes there were 180 phone calls in a day.

– LSM average size municipality

Regarding changes in orgware, the temporarily changed roles of LSMs and NSCs resulted in better contacts and collaboration with sports clubs and other local sport providers. In addition, they contacted sport providers in their municipality they were not yet familiar with. Before the pandemic, there was, for example, little collaboration with fitness and health centres.

#### Increased support for sports clubs

National politics, especially the forced closure of sports clubs and social distancing rules, enhanced existing problems of some sports clubs (at the local level) such as decreases in club members, in particular for team and indoor sports, and difficulties in finding and maintaining volunteers (problems):

Many people who participated in club sport now do an individual sport. People make more deliberate choices than before [the pandemic]. Previously, people participated in a cultural club, a sports clubs and they probably were board member somewhere. But now they say: ‘I am not doing everything anymore’. So, this will also have large negative impact on volunteering in the future. – LSM average size municipality

At the same time, the forced closure of sports clubs also made their societal role more visible, both for LSMs and NSCs (local politics):

It also enhanced the awareness of the importance of club sport and social connectedness. Not only for your physical health, but also for your mental health. People have missed that so much. And it was so bad for people to be isolated in such a way. – Coordinator of NSCs average size municipality

Regarding the orgware of sport promotion policy, support for sports clubs was already an important policy theme in most municipalities. However, due to the above-described developments, different municipalities decided to enhance this support by NSCs on topics like recruiting and maintaining volunteers, strengthening a club’s organisational capacity and (innovative) strategies for sports clubs to recruit and maintain (new) members.

#### Enhanced collaboration with non-sport organisations

As mentioned before, at the national level, the Dutch government emphasised the importance of regular sport and physical activity participation to stay healthy. This increased the awareness that ‘sport is good for health’ at all levels (national and local politics). At the local level, this helped in (re)connecting sport organisations and NSCs with non-sport organisations (e.g. health and welfare organisations, schools), such as in the existing local sports agreements (solution). Within the municipal office, the same applied for the connection between the sport domain and other policy domains (e.g. health, welfare) (solution). Nonetheless, for some municipalities ‘real’ collaboration and integrated policy implementation is still difficult:

It [the argument that ‘sport is good for health’] puts more focus on policy integration, especially regarding [general] health. And we have tried to include that in our [sport promotion] policy, but it is still very difficult to realise it nationally, in our municipality and in other municipalities. We wrote it down together, but now everything gets back to normal, this [urgency of policy integration] slowly disappears again.

– LSM large size municipality

Reflecting on changes in orgware, the increased awareness that ‘sport is good for health’ resulted in enhanced connections between sport and other sectors/domains (within the municipal office and at the local level). For NSCs, it enhanced the connection with non-sport organisations and made it easier for NSCs to initiate (sport) activities with(in) these organisations.

### Changes in organised sport activities (software)

The interview data revealed two common topics about changes in software: 1) More sport activities (using sport as a means) for specific target groups; 2) More (support of) sport activities in public space.

#### More sport activities (using sport as a means) for specific target groups

At the national level, there were signals that the COVID-19 pandemic and measures resulted in health problems of different population subgroups, not only physical health problems, like decreases in physical activity and increases in overweight, but also mental and social health problems (problem). This increase in health problems was also experienced at the local level by NSCs and within the municipal office by the LSMs (problem):

Loneliness has increased in all layers of society due to COVID-19, because there was a large reduction in social interactions and all daily structures got disrupted.

– LSM large size municipality

It should be noticed that at the time of the group interviews, most interviewees did not have actual monitor data to provide insight into the magnitude of these health problems within their own municipality.

Due to the experienced increase in health problems, COVID-19 recovery funds were made available by the Dutch government at the national level (in 2021; national politics). These consisted of an additional two hundred million euros to stimulate social and mental wellbeing and a healthy lifestyle among inhabitants. There was a specific focus on youth, vulnerable groups, and lonely older adults (see also: Dutch Government 2021). Municipalities could apply for this funding to initiate activities, including sport and physical activities, for these target groups. Most participating municipalities applied for these additional financial resources. When sport was used as a means, this often led to new local collaborative sport initiatives (solution):

In collaboration with a welfare organisation and a cultural organisation, we started a project focused on adolescents in their free time. This project was started from COVID-19 recovery funds. Within this project, a NSC and youth worker informally talk with youth about their [mental] problems through a sportive activity, such as laser gaming, soccer, and basketball. – Coordinator of NSCs large size municipality

For some LSMs, the visible health problems provided a moment of critical reflexivity considering their target groups. So, in a few cases, this led to attention for new target groups within a municipality’s sport promotion policy (solution). However, the available funds were often spent on implementing additional sport activities for existing target groups, in particular children, adolescents, and older adults (i.e. the target groups of the funding; solution). Nonetheless, municipalities were happy with the additional financial resources, because in general there is little budget for sport promotion of specific target groups:

Normally, our budget [for sport promotion of specific target groups] is limited, but now with COVID-19 there are all kinds of funding arrangements. So that makes you think: ‘how can that be?’ Suddenly, it was possible.

– NSC average size municipality

Regarding changes in software, the combination of increased experience of health problems, the awareness that sport can decrease these health problems and availability of COVID-19 recovery funds, led to more collaborative sport initiatives (using sport as a means) for children, adolescents, and older adults.

#### More (support of) sport activities in public space

We already described how the COVID-19 pandemic led to more attention for sport and sport facilities in public space (see also: *more (attention for) sport facilities in public space)*. This also resulted in NSCs (at the local level) organising more sport activities in public spaces, such as on playgrounds and in parks (solution). However, due to an increased experience of health problems with youth (problem) and the available COVID-19 recovery funds at the national level (national politics) in the second year of the pandemic (see also the previous paragraph), most additional outdoor activities were organised for this target group (solution). When sport was used as a means, there was often enhanced collaboration within the municipal office between the LSM and other policy domains (e.g. youth, education) and outside the municipal office between NSCs and other local actors (e.g. youth and health workers) to implement the activities (solutions).

Reflecting on changes in software, in some municipalities, these collaborative outdoor sport activities for youth are now organised on a (more) structural basis. Moreover, some NSCs provide (more) support to other local parties to organise these kinds of activities.

## Discussion and conclusion

As argued in the introduction, local sport promotion policy is influenced by different environmental systems (Fahlén and Stenling 2016, Vos *et al*. 2016, Hoekman and Scheerder 2021, Hoekman *et al*. 2022). In our case, we use the natural experiment of the COVID-19 pandemic to assess the role of different environmental systems in changes in local sport promotion policy. We applied the principles of the MSF within a socioecological model of local sport promotion policy to examine whether the COVID-19 pandemic created a ‘policy window’ for policy change and if so why. Our study revealed that the COVID-19 pandemic indeed opened a ‘policy window’ and resulted in local policy changes. Furthermore, linking to our first research question (why), we found evidence for the layered perspective of local sport promotion policy and the application of the MSF principles (i.e. the problem, policy/solution and politics streams) as explanation for why changes occurred. So, in line with previous studies (Fahlén and Stenling 2016, Vos *et al*. 2016, Hoekman and Scheerder 2021, Hoekman *et al*. 2022), we conclude that local sport promotion policy is situated in and impacted by a broader context. As such, the applied theoretical framework was purposeful for the analysis. The MSF principles showed that the COVID-19 pandemic and following national politics impacted the local sport promotion policy through the different environmental systems (i.e. the local external drivers and internal drivers). We hypothesised that the COVID-19 pandemic would further enhance the shift to a focus on the instrumental values of sport and would create a favourable (policy) environment for sport promotion policy. Indeed, the results indicate that national politics influenced local politics and, as such, enhanced the awareness of the importance of sport for health. Consequently, this argument was used at all levels to make sport’s case and legitimise the existence of sport promotion policy and sport promotion activities for a broader purpose (Strittmatter *et al*. 2018, Stenling and Sam 2019).

Regarding our second research question (how), we hypothesised that the enhanced focus on the instrumental values of sport would lead to changes in the sport promotion policy related to the facilities and public space (hardware), organisational actors (orgware) and organised activities (software). We indeed observed changes in policy activities. In most cases this did not refer to totally new policy activities, but merely a modification of existing activities linked to developments in the environment and shifts in the perceived importance of policy themes (i.e. local politics). A clear example is the increased attention paid to sport facilities in public space (hardware). Before the pandemic, municipalities already paid attention to healthy and physical activity friendly environments (Ruijgt *et al*. 2018, Noordzij *et al*. 2020). However, at that time LSMs were hardly involved in developing these plans (Noordzij *et al*. 2020). During the COVID-19 pandemic the public space became more important for sport participation, physical activity and for a resilient population (Grubben *et al*. 2022). Other policy domains (health, urban planning) became more aware of the meaning of public space and more specific the physical activity friendly environment for physical activity and health promotion.

Furthermore, the increased support for sports clubs stands out (orgware). Sports clubs have a key role in sport promotion. The COVID-19 pandemic accelerated existing problems of sports clubs such as decreases in members and volunteers. National data showed that 27% of the Dutch sports club members of 2019 dropped out by 2021 (Hoekman *et al*. 2023). In line with the MSF principles, the COVID-19 pandemic made these problems more visible and urgent so that they became issues that needed to be addressed on the policy agenda. As a consequence, LSMs and (coordinators of) NSCs felt the need to support and revitalise sports clubs more intensively during the COVID-19 pandemic. This is also due to the policy relevance ascribed to sports clubs as important implementers and ‘translators’ of local sport promotion policy (Skille 2008, Van der Werff *et al*. 2015) and the political debates on the need to keep sports clubs afloat. Furthermore, when policy makers want to use sport to reach broader aims, such as health and welfare, it is important that sports clubs have the skills and capacity to realise these aims. It is not self-evident that sports clubs will contribute to these policy aims and accept this responsibility (Stenling 2014, Fahlén *et al*. 2015). For this, it is important to involve sports clubs in the policy process, align with their needs and ambitions, and support them on realising broader health aims (Ooms 2020, Stenling and Sam 2020). Although sports clubs and other relevant stakeholders have become more actively involved in local sport promotion policy making (e.g. with the local sports agreements), this remains a point of concern (Ooms 2020, Hoogendam *et al*. 2021).

With respect to changes in software, the COVID-19 pandemic and resulting national politics (i.e. the available COVID-19 recovery funds) enhanced the implementation of targeted sport promotion activities (i.e. for children, adolescents, and older adults). However, from the introduction, it is shown that particular people, those with low incomes, financial problems, low education and the unemployed, decreased their sport and physical activity behaviour during the pandemic (De Boer *et al*. 2021, Grubben *et al*. 2022, Kyan and Takakura 2022, Widyastari *et al*. 2022). These subgroups were not mentioned in the interviews and only to a lesser extent in the questionnaire data. Furthermore, the interview data showed that LSMs did not have recent monitor data to confirm decreases in sport and physical activity behaviour or increases in health problems within specific subgroups. Considering the increased social inequalities in sport and physical activity participation, it is important that municipalities pay attention to these groups and gather monitoring data to ensure sport promotion policy activities are aimed at the right target groups (Ooms 2020). If the latter is not the case, social inequalities may be further widened and deepened by the policy measures taken (Collins and Haudenhuyse 2015).

This study provides a better understanding of the dynamics of local sport promotion policy making, using the MSF principles within the different environmental systems that influence local sport promotion policy. In addition, this study points to several avenues for further research. First, future research is needed on the extent to which policy changes due to the COVID-19 pandemic are maintained and implemented at the local level, and on whether the changed policy is effective in reaching health-related aims and decreasing social inequalities in sport and physical activity participation. Second, although we included the ‘policy entrepreneurs’ with regard to local sport promotion policy development in the Netherlands (i.e. the LSMs and (coordinators of the) NSCs), a broader consultation in the future including local sport providers and representatives of other policy departments, is likely to bring additional perspectives on the contextual drivers on local sport promotion policy. Third, this study included only a limited sample of municipalities in the qualitative study. Although we tried to get a diverse sample based on the online questionnaire responses, in all 11 included municipalities changes in local sport promotion policy due to the COVID-19 pandemic occurred. So, it is possible that there was some selection bias in the sense that only LSMs that were interested in the topic and/or municipalities where the pandemic was relevant for sport promotion policy responded to our invitation for a group interview. Nonetheless, the results do provide insight into why and how an external focusing event, such as the COVID-19 pandemic, can influence local sport promotion policy. Finally, a case study design was used focusing on the Dutch context. We believe that the layers of the socioecological model are applicable to other countries with similar sport policy systems (e.g. Belgium, see also Vos *et al*. 2016) or can be adapted to different sport policy systems in other countries or continents by altering the environmental layers. Moreover, the generic principles of the MSF have already been proven to be applicable to a diversity of contexts and the terms describing local sport promotion policy (i.e. hardware, orgware and software) are universal and can be applied in other countries as well. Consequently, it would be interesting to apply our theoretical framework in other countries to evaluate changes in local sport promotion policies or to apply it for other focusing events such as the energy crisis, climate change, or an economic recession.

It should be noted, however, that the ability for policy entrepreneurs to make use of a ‘policy window’ is essential for policy change. When as a result of an external focusing event, a policy window opens, policy entrepreneurs must be ready to promote their problems and solutions. For this, they need to be well-placed and able to connect the three streams. This applies to the LSMs and NSCs who are strongly positioned in most Dutch municipalities. Similar officials (especially regarding the NSCs) may not be present in other countries. Furthermore, the contextual drivers may work out differently in different countries. We anticipate this to be partly dependent on the existing position of sport in local and national policy and the society as a whole, and the strength of the local sport sector. In the Netherlands, a strong discourse exists on the instrumental values of sport and the vibrant voluntary sports club structure is well connected with local sport promotion policy. This may be different in other countries (e.g. the UK; see also Collins and Haudenhuyse 2015). All in all, it would be interesting to study this in other countries as well to identify how this may work out differently in other countries based on differences in how policy entrepreneurs are placed, the contextual drivers, and the position of sport in policy and society.

To conclude, we found proof for contextual drivers on local sport promotion policy development in the Netherlands. The COVID-19 pandemic has created a ‘policy window’ for the sport sector in general and sport promotion policy in particular to be seen as a relevant partner or policy area to solve broader health issues. In addition, the pandemic focused more attention on specific sport promotion policy themes and target groups. It is important for LSMs and NSCs as policy entrepreneurs to take advantage of this ‘policy window’ and use it to create advocacy and support for sport promotion policy and sport promotion activities over a longer period (Strittmatter *et al*. 2018, Stenling and Sam 2019). With the current policy framework ‘Healthy and Active Living Agreement’ the funding for NSCs and for local sports agreements is secured for the upcoming years (Dutch Ministry of Health, Welfare and Sport 2023). As policy change and implementation normally takes a period of multiple years (Houlihan 2005) this provides (financial) opportunities for LSMs and NSCs to follow this through.

## Data Availability

The data presented in this study are available on request from the corresponding author. The data are not publicly available due to the collection method and to preserve the privacy of respondents.

## Note

1. Informed consent is obtained through the panel. Because participants are not exposed to procedures and do not have to follow certain behavioural rules, approval by a medical ethics committee is not mandatory (Dutch Government 2023).
